# Comparative non-target screening of advanced oxidation process (AOP)-integrated industrial wastewater treatment using RP and mixed-mode chromatography

**DOI:** 10.1007/s00216-026-06691-z

**Published:** 2026-07-27

**Authors:** Felix Drees, Max Reuschenbach, Gerrit Renner, Torsten C. Schmidt

**Affiliations:** 1 Instrumental Analytical Chemistry, University of Duisburg-Essen, Universitätsstraße 5, 45141 Essen, Germany; 2 Centre for Water and Environmental Research, University of Duisburg-Essen, Universitätsstraße 5, 45141 Essen, Germany; 3 LANUK Landesamt Für Natur, Umwelt Und Klima Nordrhein-Westfalen (LANUK), Leibnizstr. 10, 45659 Recklinghausen, Germany; 4 IWW Water Centre, Moritzstrasse 26, 45476 Mülheim an Der Ruhr, Germany

**Keywords:** Non-target screening, Advanced oxidation process, Industrial wastewater, Transformation products, Polarity, Chromatography

## Abstract

Advanced oxidation is increasingly integrated into industrial wastewater treatment to address complex, variable contaminant loads. Here, LC-HRMS non-target screening (NTS) was used to characterize an industrial WWTP incorporating a one-stage advanced oxidation process (AOP) and to compare reversed-phase (RP) and mixed-mode (MM) chromatography for expanding the accessible polarity window. Both methods delivered consistently high data quality, enabling robust feature-level comparisons. Cliff’s *δ* and polarity descriptors indicated differences in feature distributions, while tentative matching to the NORMAN Substance Database suggested substantial overlap between RP and MM and pointed to potential database bias toward less polar compounds. Multivariate analyses captured treatment-driven chemical patterns: PCA separated AOP-treated samples most clearly with MM, consistent with enhanced sensitivity to polar transformation products, whereas RP more strongly differentiated influent from effluent, reflecting predominantly hydrophobic signals. PLS-DA confirmed these trends and enabled robust classification of sample types. Volcano analyses highlighted statistically significant increases, decreases, and persistent signals, showing that while biological treatment removed most components, a subset of AOP-derived features persisted into the final effluent. Overall, broadening the chromatographic window improves NTS-based process assessment and supports targeted optimization of treatment steps for problematic compounds.

**Graphical abstract:**

**Figure:**
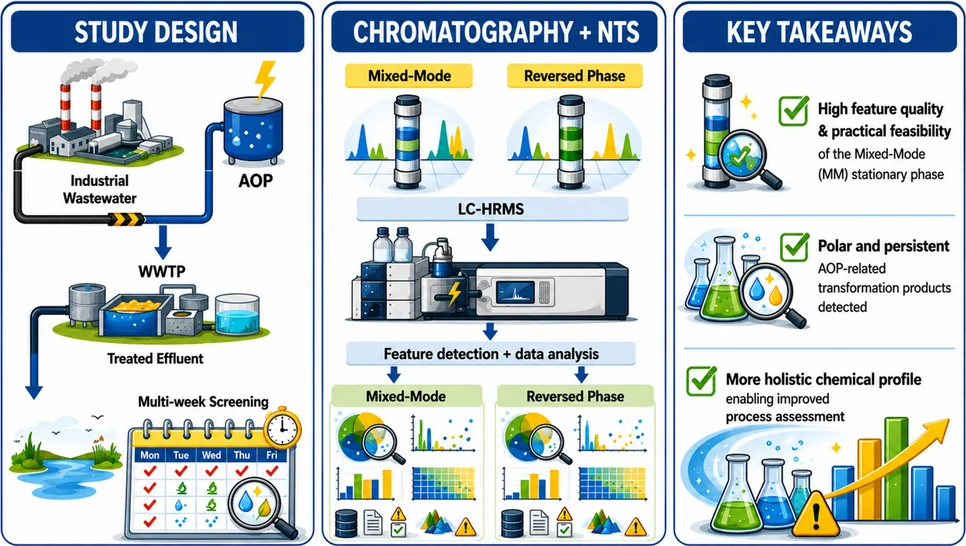

**Supplementary Information:**

The online version contains supplementary material available at https://doi.org/10.1007/s00216-026-06691-z.

## Introduction

Non-target screening (NTS) approaches aim to identify and characterize a broad range of chemical compounds without specifically targeting known substances and to potentially use this information to characterize processes. The goal is to better understand the chemical space, including unexpected or unknown compounds. This approach is valuable for gaining insights into complex chemical matrices and is particularly useful in environmental studies where a diverse array of substances may be present [1].

However, the analytical scope is limited instrumentally. One constraining factor is chromatographic separation, primarily determined by the chosen stationary phase. Typically, NTS studies utilize reversed-phase (RP) columns, which are known for their robustness. Nevertheless, chromatography is reduced to mostly non- to medium-polar substances in these cases. While some RP columns have been modified with polar end-capping to expand the analytical range, a significant portion of polar compounds remains unaccounted for, a problem that has been much discussed in the scientific community [2, 3]. This is not only because polar compounds are typically not covered, and there is likely a wide range of previously unknown pollutants within this group, but also because their physicochemical properties are particularly mobile in the aquatic environment [4]. Moreover, regulatory authorities and water suppliers show growing interest in implementing NTS approaches to polar compounds to maintain high water quality [5, 6].

Hydrophilic interaction liquid chromatography (HILIC) phases have been tested recently as a promising complementary approach to RP chromatography [6–9]. However, challenges arise, such as stability issues during the injection of water samples, solubility problems, excessive column bleeding, strong pH- and salinity dependencies, and, in this scope, the question regarding matrix robustness in general [10–13]. Moreover, a HILIC-based analytical approach alone would, in turn, discriminate against non-polar compounds. Therefore, complementary analysis using an RP stationary phase would remain essential to obtain the most comprehensive chemical profile possible. This additional effort underscores the need for a chromatographic strategy that enables the simultaneous detection of polar and non-polar compounds in a single analytical run. Although two-dimensional chromatography coupling [14] and column-switching techniques [15] address this issue, they introduce significant instrumental complexity and complicate data interpretation. Accordingly, we emphasize the role of mixed-mode (MM) phases in this study. These phases incorporate polar and non-polar interaction mechanism combinations, often realized through integrating different functional ligands on the stationary phase surface. As such, they offer the unique ability to retain a broader spectrum of analytes—potentially ranging from highly polar to non-polar compounds—within a single chromatographic run while minimizing solubility-related challenges [16]. Given the diversity of potential ligand combinations and MM types, there appears to be significant yet unexplored potential for expanding the polarity range in NTS studies [17]. In previous works, MM chromatography has already been proven to apply to surface and WWTP effluent water analysis [18, 19]. However, industrial wastewater, especially influent samples, poses a unique challenge since water matrices are highly complex and dynamic and include a wide variety of polar and non-polar organic substances. Therefore, we aim to further explore the potential applicability of MM chromatography in non-target screening studies through a multi-week analysis of industrial wastewater. Given that the existing body of research on MM chromatography in the NTS field remains somewhat limited, this study seeks insights into its effectiveness and suitability for complex environmental samples.

## Material and methods

### Study design and sampling

**Fig. 1:**
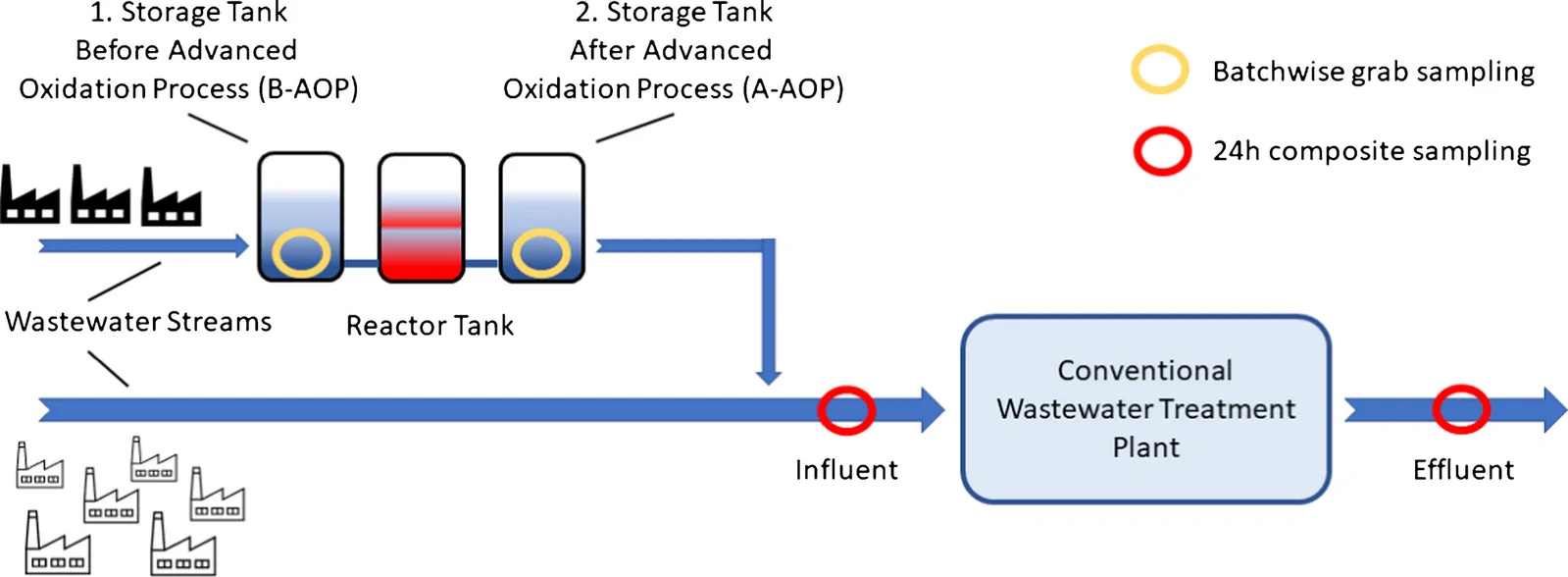
Schematic overview of sampling sites. Grab samples (marked as yellow circles) before (B-AOP) and after the advanced oxidation process (A-AOP) are withdrawn batch-wise from storage tanks. Daily composite samples (marked as red circles) are taken from WWTP influent and effluent

At the industrial site that supplied water samples, on-site wastewater purification includes an advanced oxidation process (AOP) applied as batch-wise pre-treatment to selected wastewater streams. Each batch was grab-sampled immediately before and after the AOP. Subsequently, the treated effluent merges with the main industrial wastewater flow, which undergoes biological purification at a conventional wastewater treatment plant (WWTP). Over a 24-h cycle, composite samples were collected from the WWTP influent and effluent (Fig. 1). Sampling was conducted over a total period of 9 weeks.

The samples were collected weekly, prepared, and subsequently analyzed, resulting in a maximum storage duration of up to 7 days for individual samples. Throughout the entire period, the samples were stored under cooled conditions. Initially, gravity sedimentation was allowed to occur, after which the supernatant was carefully collected and diluted for further analysis. Samples were analyzed by direct injection without prior extraction or enrichment. Diluted supernatant samples were injected directly into the LC-HRMS system. Quality control (QC) samples were analyzed in replicate, while individual environmental samples were injected once.

### Instrumentation

Chromatographic separation was performed on a Dionex UltiMate 3000 HPLC system (Thermo Scientific, Bremen, Germany). On the one hand, a mixed-mode (Primesep 200, 3 µm, weak acidic ion-pairing 2.1 × 100 mm + 2.1 × 5 mm guard column, SIELC Technologies, Wheeling, IL, USA) and, on the other hand, a polar end-capped C_18_ phase (XSelect® 2.5 µm HSS T3 2.1 × 100 mm + 2.1 × 5 mm guard column, Waters, Dublin, Ireland) were utilized. The HPLC system was coupled to a high-resolution mass spectrometer (HRMS) (QExactive Thermo Scientific, Bremen, Germany). Compounds were ionized by electrospray ionization (IonMax with H-ESI II probe, Thermo Scientific, Bremen, Germany) separately in negative and positive modes. Subsequently, HRMS analysis was performed in MS^1^ full scan spectra acquisition mode (*m*/*z* 80–1000) with a resolution of 70,000 at 200 *m/z,* and data-dependent MS^2^ scans with the five most intense peaks per mass spectrum with a resolution of 17,500 at 200 *m/z*. Detailed information can be found in SI Tables 1 and 2.

A blank sample was measured between all samples to prevent and detect potential carry-over events. Additionally, triplicate quality control (QC) samples were analyzed in positive and negative ionization modes at the beginning of each batch to ensure analytical consistency and reliability. These QC samples consisted of pure water spiked with a mixture of 16 known standards (1 µg L⁻^1^), serving as internal controls to monitor retention time stability, signal intensity, and overall system performance throughout the analysis. While feature quality in this study was primarily evaluated using the DQS-based workflow specifically designed for NTS data, additional analytical quality assurance metrics, including retention time reproducibility, internal standard signal stability, and matrix-related effects, are summarized in the Supporting Information (SI Tables 5–8).

**Fig. 2:**
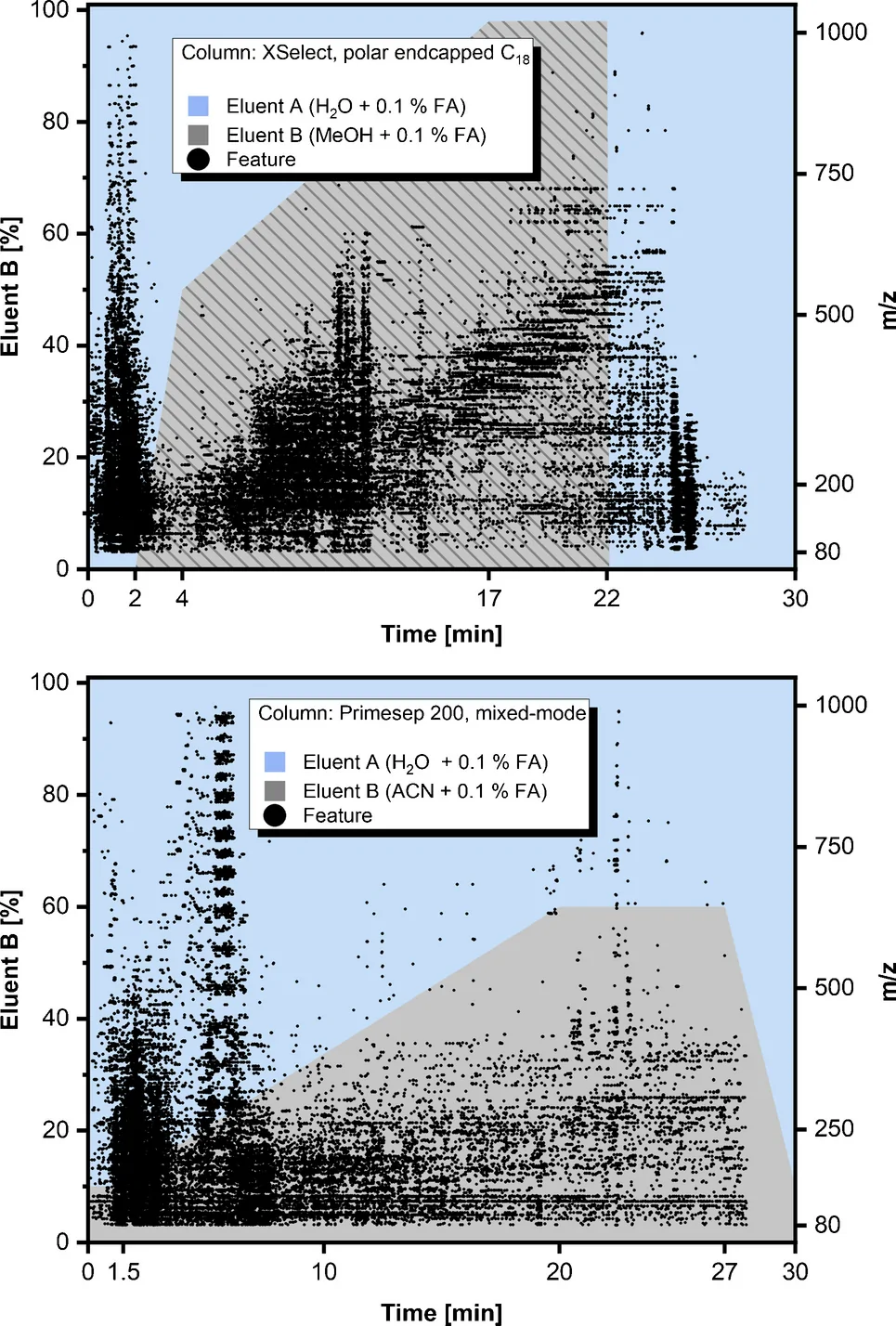
The elution profiles of the RP and MM chromatography. Overlaid on these profiles are scatter plots of the respective features (dots), illustrating their chromatographic distribution

The MM chromatographic method was operated at a flow rate of 0.3 mL min⁻^1^ with a column temperature of 20 °C. The gradient profile and mobile phase composition are described in detail to facilitate reproducibility of the method (Fig. 2).

### Non-target screening

All raw chromatographic data were acquired in profile mode using Xcalibur software (Thermo Fisher Scientific). These chromatograms were then converted to.mzML format using the msConvertGUI software (SI Table 3) [20]. The non-target screening data processing was realized using MZmine4.5.0 [21].

#### MZmine4.5 parameters

The MZmine4.5 parameters were configured according to the guidelines and preliminary recommendations proposed by the software tool developers for LC–MS data analysis [22–24]. In total, 201 samples were processed, while the workflow included mass detection, chromatogram building and deconvolution, isotope considerations, alignment, and gap-filling. Concerning system blanks, we decided to apply a blank subtraction with a fold change threshold of 200%. Here, we considered 48 system blanks. Detailed information on software settings is listed in SI Table 9. For subsequent statistical investigations, peak area information was considered.

### Statistical and multivariate analyses for assessing data differences

#### Cliff’s *δ*

In this study, Cliff’s *δ* quantifies differences between feature lists from two chromatographic conditions by calculating the *m*/*z* difference for every possible pair of features. Differences smaller than 0.005 Da are treated as zero; each pairwise comparison yields + 1 (positive difference) or −1 (negative difference). Cliff’s *δ* is then computed as the average of these values over all pairs, producing a score between −1 and 1. The magnitude and sign of *δ* indicate the effect size and direction based on established thresholds [25].

#### Multivariate analysis (PCA and PLS-DA)

Statistical assessments of the NTS data matrices from MZmine 4.5.0 were conducted by analyzing peak areas following initial data pre-processing steps, including logarithmic transformation, total area normalization, and mean-centering. As a first explorative step, principal component analysis (PCA) was applied to compare and understand the inherent structure of the NTS datasets. PCA scores enabled a visual comparison of the samples according to the experimental design characteristics. Potential outliers were assessed using *Q* residuals reduced and Hotelling’s *T*-squared reduced plots at a significance level of *p* = 0.950.

To complement PCA, partial least squares discriminant analysis (PLS-DA) was subsequently performed to highlight systematic differences between predefined sample classes. Guided by the PCA results, PLS-DA models were calculated based on groupings reflecting the experimental design. This supervised method allowed for a focused dataset assessment of class separation patterns. PLS-DA model validation was performed via cross-validation using random sampling with 10 splits and 5 iterations to assess the robustness of class discrimination.

#### Volcano plots

First, the mean values for each sample group are computed. The fold change between these groups is calculated on a logarithmic scale (log2). A *t*-test is performed for each feature to assess statistical significance, and the resulting *p*-values are adjusted for multiple comparisons using the false discovery rate method. Significant features are then identified based on an adjusted *p*-value threshold. Features were considered statistically significant when the adjusted *p*-value (FDR) was below 0.05, and the absolute log₂ fold-change exceeded 1 [26]. The *p*-values are transformed into a −log10 scale, and the volcano plot is generated by plotting the fold change against the transformed *p*-values.

### Software

The PLS Toolbox 8.9.1 (Eigenvector Research Inc., Wenatchee, WA, USA), working under MATLAB (The Mathworks, Natick, MA, USA), performed PCA and PLS-DA. Generally, figures were designed with OriginLab (Pro), version 2024b. To create volcano plots, the App “VolcanoPlot” in version 1.10, which is developed by OriginLab Technical Support and freely available at https://www.originlab.com/fileExchange/details.aspx?fid=296, was applied, while the statistical calculations were implemented in the MATLAB environment.

## Results and discussion

### Chromatography

Generic gradient methods are commonly applied in the NTS field [10] to comprehensively capture a wide range of compounds. Here, RP chromatography was performed using established in-house conditions based on methanol and water, which have been routinely applied in NTS studies within the research group for several years [26–29]. A generic gradient using acetonitrile and water was applied for the MM stationary phase, following the manufacturer’s recommendations for complex sample matrices [30]. In both cases, eluents were acidified with 0.1% formic acid, the ionization and HRMS conditions were standardized, and the analysis time was 28 min. Figure 2 presents a comparative overview of both gradient methods. Additionally, the respective scatter plots of the extracted features are displayed. In total, 56,121 and 31,373 features were extracted with MM and RP stationary phases. It should be noted that an increased number of detected features does not necessarily correspond to an equivalent increase in meaningful analytical information. This difference reflects the broader feature space captured by the MM approach, which is consistent with its ability to retain compounds across a wider polarity range. At the same time, the increased number of detected features may also include redundant signals, low-intensity features, or noise-related contributions, highlighting the importance of subsequent filtering and data quality assessment.

An analysis of both scatter plots reveals that, aside from the void volume, the density of extracted features remains relatively evenly distributed across the entire chromatographic range under both conditions, as intended. While some areas of increased feature density are observed—specifically, a high concentration of features around 10 and 25 min for the RP phase and around 5 min for the MM phase—the overall chromatographic performance ultimately depends on the quality of the extracted features, including chromatographic peak shape, signal-to-noise ratio, and data quality metrics summarized in the DQS.

To assess the similarity between both feature lists under the respective chromatographic conditions, Cliff’s *δ* was calculated based on the corresponding *m*/*z* values. Cliff’s *δ* is a non-parametric effect size measure that quantifies the degree of difference between two distributions. It ranges from −1 to 1, where 0 indicates complete overlap and values closer to ± 1 indicate stronger separation. According to commonly used interpretation thresholds proposed by Macbeth et al. [25], a value of approximately 0.2 corresponds to a small-to-moderate effect size, indicating a modest but systematic difference between the compared distributions. In this context, the calculated value of 0.2 suggests a modest yet systematic difference between the *m*/*z* distributions of the RP and MM feature sets. Specifically, it indicates that the molecular masses of features detected under RP conditions tend to be slightly higher than those detected using the MM stationary phase.

This result is consistent with the visual impression from Fig. 2, where the scatter plots suggest that *m*/*z* values associated with the MM phase are generally lower than those from the RP phase. This trend aligns with expectations, as polar compounds—preferentially retained by MM stationary phases—typically have molecular masses below 400 Da [31, 32].

### Feature quality

**Fig. 3:**
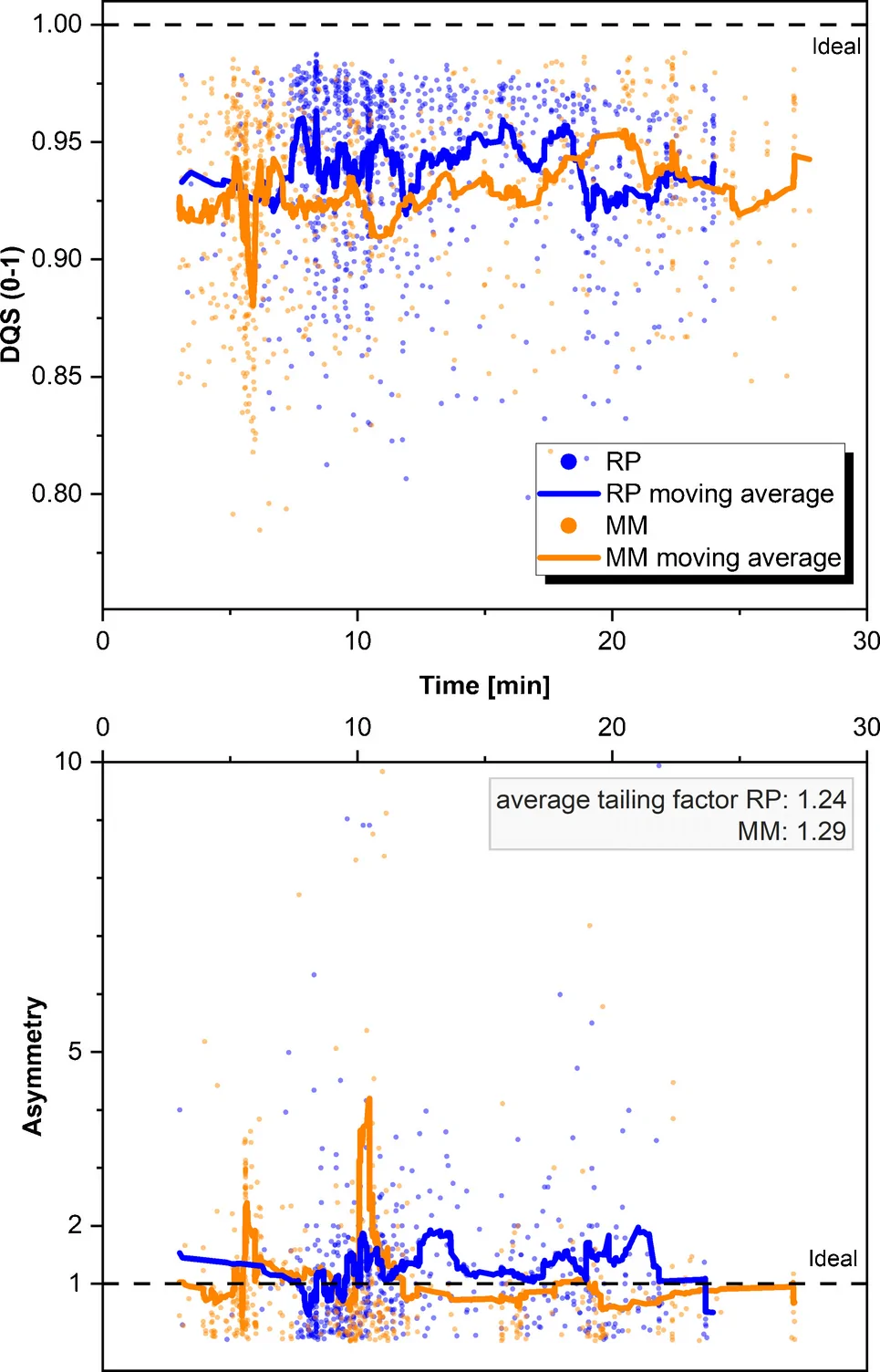
Data quality score (DQS) and asymmetry factor of extracted features as a function of their retention time (dots). Features detected using the RP are shown in blue, while those from the MM phase are displayed in pink. The corresponding moving mean (10 data points) for each approach is represented by a solid line. Since the RP chromatography involved a re-equilibration with pure water, quality metrics are only considered for up to 24 min

To assess the quality of the features, we compared their data quality scores (DQS) across both chromatographic approaches using qAlgorithms, which represents a workflow for centroiding, binning, and peak detection that was specifically developed for NTS data. The DQS is based on user-parameter-free NTS data processing [33]. It incorporates key processing steps, including centroiding [29] binning [34] and a feature’s subsequent peak detection. The final DQS is derived as an equally weighted combination of the three individual scores: DQS_cen_, DQS_bin_, and DQS_peak_. Each DQS contains information about the reliability of the results. Within the MZmine workflow, peak asymmetry was assessed as a feature-level quality descriptor based on the ratio between the distances from the peak apex to the left and right peak flanks at 10% peak height. This metric was used to evaluate whether chromatographic peak shape contributed to reduced feature quality across the retention time range. To provide an additional chromatographic reference, the average tailing factors reported by MZmine are indicated in the lower panel of Fig. 3 for RP and MM, respectively. Since chromatographic performance and coelution effects may vary across the chromatographic run and may affect signal quality in information-dense regions, both DQS and peak asymmetry are shown as a function of retention time.

The figure demonstrates that the DQS values of features across the retention time dimension are predominantly aligned with their respective ideal benchmarks, indicated by the dashed reference lines. This suggests that the overall data quality is robust and sufficiently high across both chromatographic conditions. The average tailing factor is similar in both scenarios, with values of 1.24 and 1.29, respectively, indicating a slight tailing of the peaks overall. Throughout nearly the entire chromatographic range, the feature quality, averaged using the qAlgorithm, remains above 0.9, meeting the quality standards required by the algorithm.

Regarding MM chromatography, two short intervals stand out in which the DQS moving average temporarily decreases—approximately 6 and 11 min. Simultaneously, the asymmetry factor indicates a decline in data quality. The RP chromatography can observe a similar pattern at 12 and 20 min. Although both evaluation criteria originate from different NTS algorithms, they consistently reflect similar trends in data quality assessment. This further supports the notion that integrating data quality evaluation steps into NTS workflows will contribute to improved harmonization in the future, both at the chromatographic level and in data processing [35, 36].

### Polarity

A comparative database screening was performed using the NORMAN Substance Database (May 13, 2025), incorporating compounds listed under *Drinking Water*, *Surfactants*, *Persistent*, *Mobile and Toxic Substances*, and *Industrial Chemicals*. From these categories, 4905 unique compounds were associated with monoisotopic masses within the *m*/*z* range of 80–1000, which were used for comparison with the detected features. The lower *m*/*z* limit of 80 was selected to minimize the inclusion of background signals and solvent-related ions, which are typically abundant in the low-mass region and may compromise data quality in non-target screening workflows [10].

The masses of the features were assumed to be [M + H]^+^ as the dominant species in positive electrospray ionization mode and [M − H]^−^ in negative mode. These were then matched to entries in the NORMAN database within a specified mass accuracy of 0.005 Da. As a result, 3562 tentative identifications were obtained for the RP and 2629 for the MM chromatographic separation. Among these, 1881 compounds were shared between both modes, while MM chromatography contributed 748 unique identifications not detected under RP conditions. Conversely, 1681 compounds were exclusively observed in RP, indicating broader coverage by this stationary phase for these sample types.

The distribution of estimated log *K*_OW_ values was analyzed based on boxplot statistics to evaluate potential differences in compound polarity between the two modes. The interquartile range (25th–75th percentile) of log *K*_OW_ values spanned approximately − 2.5 to 8.5 for RP and − 3.0 to 7.5 for MM. While an extended polarity range would be expected for the MM phase based on its mixed-mode retention mechanisms, this trend is not reflected in the log *K*_OW_ distributions derived from tentative database annotations. This apparent contradiction can be attributed to limitations of the database used for tentative identification. The NORMAN Substance Database is biased toward less polar compounds, which limits the annotation of highly polar features and results in their systematic underrepresentation in the log *K*_OW_ analysis. Independent of database-based annotations, the broader polarity coverage of the MM phase is supported by its chromatographic behavior. The distribution of detected features across the retention time dimension and the observed shift toward lower *m/z* values suggest the presence of more polar compounds, which are typically not retained efficiently under RP conditions. These observations are consistent with established retention mechanisms of mixed-mode stationary phases. A plausible explanation lies in the database, which predominantly contains compounds with log *K*_OW_ values between − 4 and 9 (SI Fig. 4). This limited range introduces a systematic bias that hampers the identification of more polar substances and likely results in their underrepresentation.

Nevertheless, the MM phase demonstrated a substantial overlap with the chromatographic space of RP, indicating that it effectively captures at least a comparable and partially complementary range of analytes.

However, the true advantage of polarity extension becomes evident when examining samples from the AOP treatment more closely. During the AOP, the formation of more polar transformation products is generally expected compared to their respective parent compounds. Since the identification of such transformation products is inherently limited by the RP-dominated reference database, we aimed to assess this indirectly by correlating potentially formed transformation products with parent compounds using Pearson correlation analysis (Pearson coefficient ranging from − 1 to + 1, with − 1 indicating strong anticorrelation and + 1 strong correlation). The correlation-based approach is applied both to assess feature continuity between treatment stages and to evaluate overlap between chromatographic methods, providing a unified framework for feature-level comparison.

**Fig. 4:**
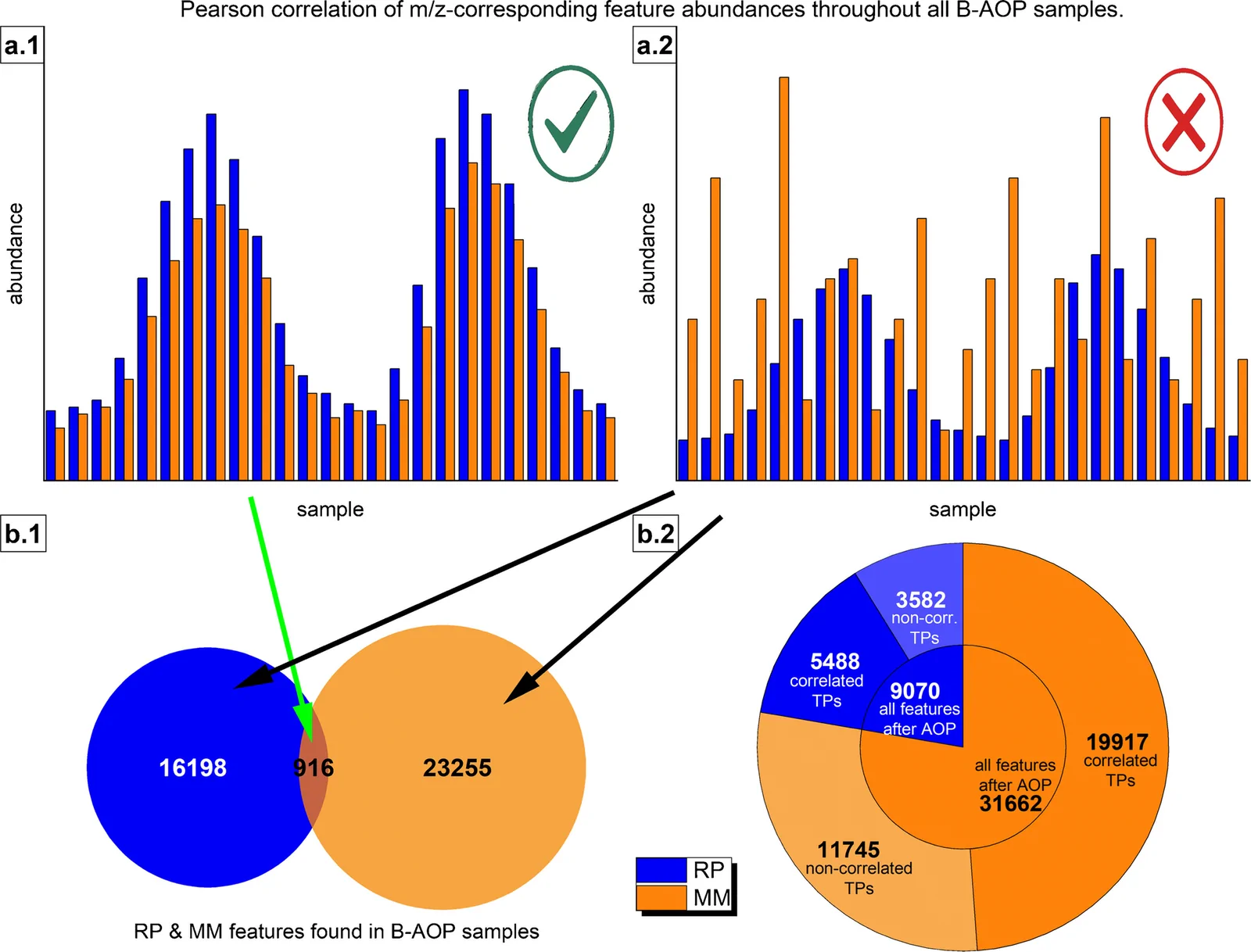
Correlation-based feature matching and assessment of potential transformation products (TP candidates). **a.1** and **a.2** Examples of a correlated and a non-correlated feature pair, respectively, by depicting abundance profiles across all samples prior to AOP treatment (B-AOP). Correlated features exhibit similar abundance trends across both chromatographic modes, suggesting correspondence. **b.1** A Venn diagram summarizing features from B-AOP samples detected using RP and MM stationary phases, respectively. A total of 916 features were identified as matching in both *m*/*z* (within 0.005 Da) and abundance profiles (Pearson correlation coefficient > 0.9) and are thus considered shared between the two chromatographic modes. **b.2** A sunburst diagram depicting all features subsequent to the AOP (A-AOP) detected by each chromatographic mode and subdivides them into potential TP candidates (identified by correlation between B-AOP and A-AOP samples) and non-correlated features

To this end, we defined a joint chemical space based on RP- and MM-associated features detected in the samples before the AOP (B-AOP). Features were considered matched at the feature level if they agreed within a mass tolerance of 0.005 Da in the *m*/*z* dimension and exhibited a strong correlation (Pearson coefficient > 0.9) across the 25 B-AOP samples between RP and MM detection. It should be noted that this matching strategy does not constitute compound identification. Correlated features may represent different isobaric compounds that co-vary due to shared sources or process dynamics and should therefore be interpreted with caution. This limitation is inherent to MS^1^-based non-target screening workflows, where feature matching relies on mass accuracy and statistical relationships rather than structural confirmation. While the combination of mass agreement and strong correlation reduces random associations, it cannot fully exclude false-positive matches. Therefore, the matching approach applied here is intended for exploratory, feature-level comparison of chromatographic behavior rather than for establishing chemical identity. Further confirmation would require additional evidence, such as MS^2^-based annotation or targeted analysis. To illustrate this aspect, examples of both a correlated and an uncorrelated feature pair are shown in Fig. 4a.1 and a.2. These panels depict the respective abundance profiles of selected features across all B-AOP samples. The differing abundance trends clearly indicate the presence or absence of correlation. Ultimately, a total of 916 feature pairs fulfilled both selection criteria—*m*/*z* agreement and consistent abundance correlation—highlighted in panel b.1. Given the harsh conditions of the AOP treatment, transformation products are expected to form for each parent compound, which again should be reflected by a strong correlation with the respective parent signals. Accordingly, the 25 B-AOP samples were compared to corresponding samples after the AOP (A-AOP) to detect potential transformation products showing a Pearson correlation coefficient > 0.9 (panel b.2).

Using RP chromatography, 5488 correlated features were identified, whereas MM chromatography revealed 19,917 correlation-based TP candidates. It should be emphasized that these numbers represent an upper estimate derived from a screening-level approach and do not correspond to confirmed transformation products. It should be noted that correlation-based identification of transformation products is inherently limited, as correlated features may also arise from co-varying matrix components or general environmental trends. Consequently, the number of detected TP candidates likely represents an overestimation of actual transformation products. The approach applied here is therefore intended as a prioritization step within the NTS workflow, highlighting features of potential interest for subsequent identification using orthogonal evidence such as MS^2^ spectra, structural annotation, or targeted experiments. The higher feature count observed for MM is consistent with its broader polarity coverage, yet it should be noted that an increased number of features does not necessarily correspond to an equivalent increase in meaningful analytical information.

In contrast, RP chromatography captures only a fraction of the potential transformation products in this context, suggesting a significant limitation in its applicability for detecting polar degradation products. This underscores the value of MM chromatography as a powerful alternative for broad-scope non-target screening. Importantly, the MM phase employed here—featuring long hydrophobic ligands combined with weakly acidic functional groups—is only one of many possible ligand configurations available in MM stationary phases. Consequently, the potential applications and advantages of MM chromatography extend far beyond this specific example, highlighting the vast and largely untapped possibilities for tailoring MM phases to cover an even wider range of polarities. This positions MM chromatography as a highly versatile and promising approach for future analytical developments.

### Treatment process characterization

**Fig. 5:**
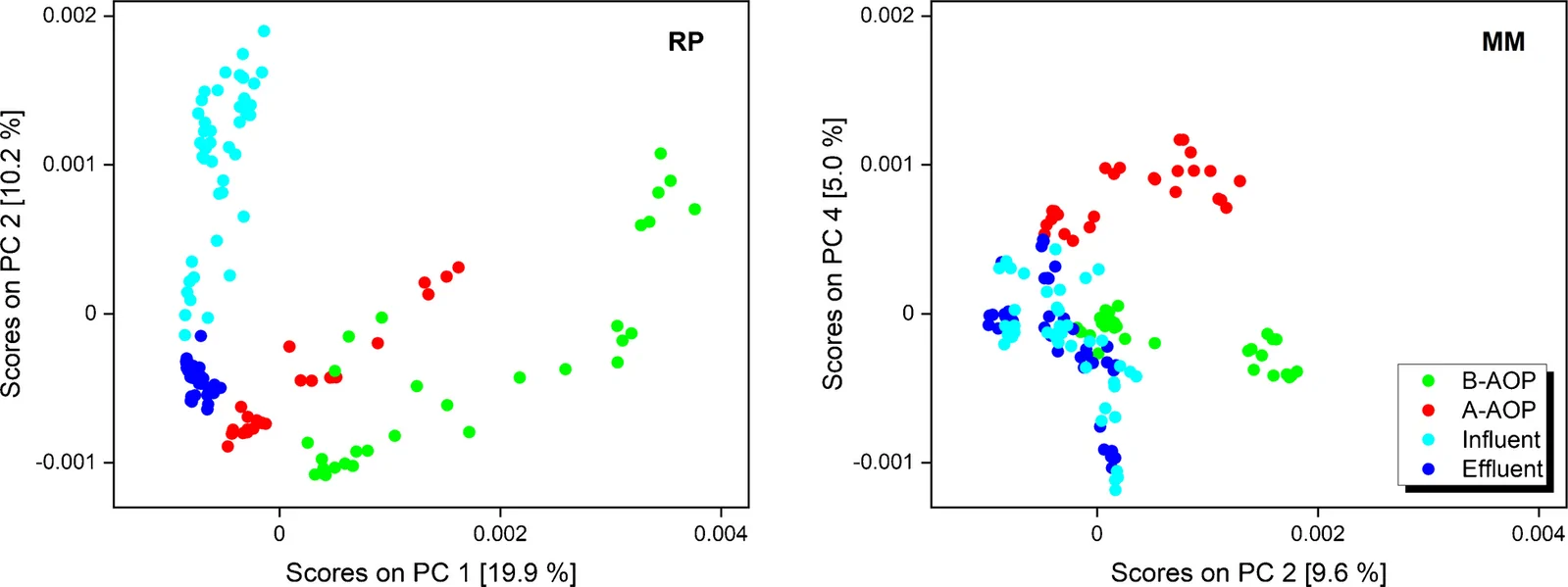
Principal component analysis (PCA) plot representing sample distribution regarding sample type differentiation. The two PCA plots, attributed to the two stationary phases, illustrate the distribution of samples based on their principal components, providing an overview of variance and clustering patterns. The *x*-axis and *y*-axis represent the combination of two principal components, which capture the highest variance regarding the sample type differentiation in the dataset. Each point corresponds to an individual sample, with color codes distinguishing sample groups. The relative distances between points indicate similarities or differences in their overall composition, with closer points representing samples that are more similar

The NTS datasets were exploratorily analyzed using PCA. Given the largely unknown nature of the datasets, the initial focus was on differentiating the samples based on their types (B-AOP, A-AOP, influent, effluent). The datasets were represented in individual comparisons of principal components. Figure 5 presents the most informative score plots for log-transformed, normalized, and mean-centered data. Samples collected before and after the AOP could be clearly distinguished based on their distribution along the principal components under chromatographic conditions. However, differentiation between the influent and effluent of the conventional wastewater treatment plant is only evident in the RP-associated data. For the MM data, no principal component combination could distinguish between these two sample types.

Differentiation between individual sample types, especially those collected in chronological sequence, relies on the assumption that their chemical profiles change significantly over time. If no separation is observed in the PCA, as seen for influent and effluent samples analyzed using the MM phase, this may suggest that the overall chemical composition remains unchanged during treatment, or that a considerable portion of substances is unaffected by the process. A similar interpretation can be made for samples located in the transitional area between groups, as observed for influent and effluent samples in RP chromatography, further supporting the notion of minimal compositional change.

Considering the dynamic nature of industrial wastewater composition, which is heavily influenced by production cycles and the specific input of various industries, influent samples located close to effluent samples in the PCA score plot may reflect wastewater streams containing a large proportion of compounds that are only minimally degraded—or not degraded at all—during treatment. In contrast, influent samples positioned further away from effluent samples in the multivariate space may indicate particularly high contamination events, as suggested by their distinct chemical signatures. Such samples may be particularly interesting for targeted follow-up, for instance, in toxicological evaluations, where elevated pollutant loads are of greater concern. The relatively low explained variance reflects the high chemical complexity and temporal variability of the dataset, limiting the interpretability of PCA for classification purposes, while still providing insight into the overall structure of the data.

While the PCA provided valuable initial insight into the chemical diversity and potential grouping patterns within the sample sets, it is essential to emphasize that PCA remains an unsupervised, exploratory technique. Consequently, it does not assess classification performance or provide definitive evidence for sample discrimination. To address this, partial least squares discriminant analysis (PLS-DA) was applied as a supervised modeling approach to quantitatively evaluate the classification accuracy between sample types, particularly concerning their chronological progression through the treatment process. This approach enables a more targeted assessment of the chromatographic information content, allowing for direct comparison between RP- and MM-based data regarding their ability to distinguish between critical sample classes, such as B-AOP and A-AOP, as well as influent and effluent of the wastewater treatment plant.

Both the RP- and MM-based PLS-DA models successfully discriminated between samples collected before and after the AOP process, reflecting the substantial chemical transformations induced by oxidative treatment. Permutation testing (*n* = 1000) confirmed the statistical significance of all models (Rand *t*-test, *p* ≤ 0.005). A detailed overview of model performance, including sensitivity, specificity, classification error, RMSECV, and R^2^CV for calibration and cross-validation, is provided in SI Table 10.

Overall, the MM-based model showed slightly superior classification performance for samples collected before and after the AOP process, exhibiting fewer classification errors and improved predictive performance during cross-validation. In contrast, the RP-based model more reliably discriminated between wastewater treatment plant influent and effluent samples. Furthermore, probability plots demonstrated that the MM-based model correctly classified several ambiguous AOP-related samples that remained uncertain in the RP-based model (SI Fig. 6).

These findings align well with the hypothesized polarity-driven behavior of compounds across treatment stages and chromatographic phases. The superior performance of the MM-based model in classifying AOP-related samples likely reflects its enhanced capacity to capture a broader spectrum of polar transformation products generated under oxidative conditions. In contrast, the more reliable classification of influent and effluent samples by the RP-based model indicates that the observed differences in classification performance are primarily driven by stronger changes in the hydrophobic fraction of the chemical space, whereas more polar compounds exhibit more similar behavior across influent and effluent samples. These findings indicate that the observed differences in classification performance are primarily driven by underlying chemical dynamics during the treatment process rather than the analytical superiority of one chromatographic approach. In particular, hydrophobic compounds, which are preferentially captured by RP chromatography, appear to undergo more pronounced changes during biological treatment, leading to clearer separation between influent and effluent samples. In contrast, more polar compounds detected by the MM approach tend to exhibit more similar behavior across these treatment stages, resulting in reduced class separability. This highlights that RP and MM chromatography provide complementary perspectives on the chemical space, reflecting different transformation behaviors of compound classes rather than differences in analytical performance.

**Fig. 6:**
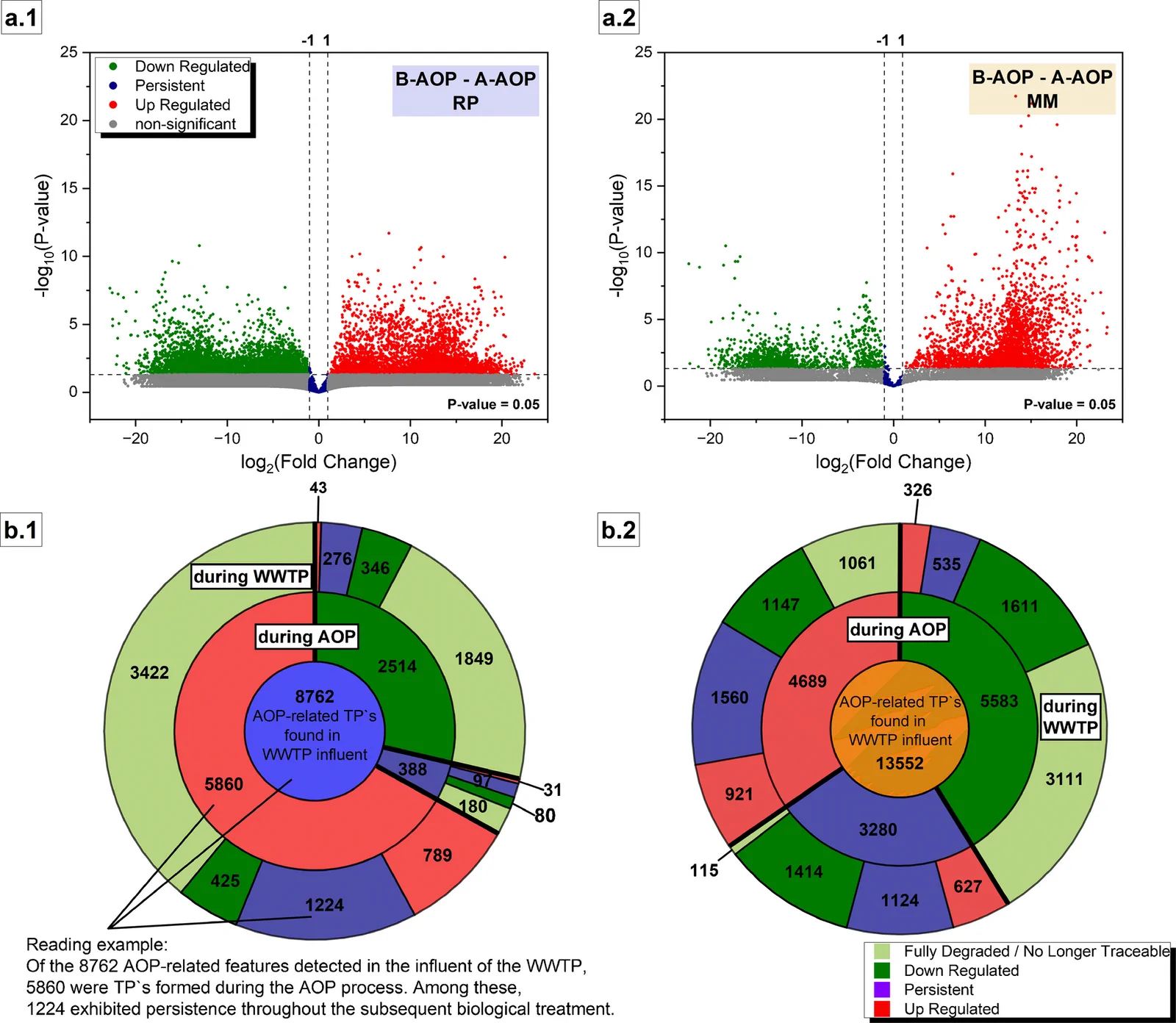
Volcano plots (**a.1**, **a.2**) illustrating statistically significant changes in feature abundance during the AOP process, with degraded features shown in green, transformation products in red, and persistent features in blue. For completeness, we also performed a volcano analysis on the combined RP and MM NTS datasets for the AOP process and the WWTP, as shown in SI Fig. 5, providing a holistic view of the NTS data. Corresponding sunburst diagrams (**b.1**, **b.2**) summarize the subsequent fate of these AOP-related features during biological treatment, using the same color code with the addition of light green to indicate features no longer traceable (completely removed). The concentric structure of the sunburst diagrams enables stepwise classification of feature persistence, degradation, or transformation during treatment

These initial observations are further supported by the monitored changes in individual feature abundances, as illustrated in the volcano plots (Fig. 6). Depending on the chromatographic phase, features that either show statistically significant changes during the AOP process or remain unaffected are highlighted in color (panels a.1 and a.2). Features depicted in green on the left side of the graphs represent degraded compounds, while transformation products are shown in red on the right. Persistent features (blue) are located in the center of the plots.

The same color scheme is applied in the subsequent sunburst diagrams (panels b.1 and b.2), which summarize the fate of these AOP-related features during the following biological treatment. In addition, features shown in light green represent those no longer traceable, i.e., completely removed. The concentric structure of the diagrams enables a stepwise classification of features according to their persistence, degradation, or transformation during subsequent biological treatment.

In general, the data indicate that most features qualitatively decrease in intensity or are eliminated throughout the wastewater treatment process. It should be noted that these percentages are derived from relative feature intensity changes observed in the non-target screening data and therefore do not represent absolute quantitative removal efficiencies of individual compounds. Regarding the WWTP effluent, 72% of features detected by RP chromatography were significantly reduced or completely eliminated, compared to 62% for MM chromatography. More specifically, 62% (RP) and 32% (MM) of features were no longer traceable, either because they were fully degraded or had fallen below the detection limit due to dilution with the bulk wastewater stream.

Considering the further fate of features already down-regulated during the AOP process, both chromatographic approaches reveal a consistent trend: approximately 90% of these features continued to decline or were eliminated during biological treatment. Features that persisted during the AOP process were largely removed in the WWTP, with only one quarter to one third remaining detectable. A striking difference between the two chromatographic phases, however, lies in the absolute number of persistent features. While RP chromatography captured 388 such features, MM chromatography identified more than an order of magnitude higher, namely 3280. This highlights a clear analytical gap that can be closed by employing MM chromatography, particularly since persistent features are of special interest with respect to potential ecotoxicological relevance. If such features were associated with adverse environmental effects, this would necessitate an adaptation of wastewater management strategies.

Transformation products generated during the AOP process and their subsequent behavior during biological treatment are of particular interest, especially when considering the extended polarity window captured by MM chromatography. Based on the volcano analysis, 5860 (RP) and 4689 (MM) features were classified as TP candidates, defined as features showing statistically significant increases in A-AOP compared to B-AOP samples according to predefined fold-change and adjusted *p*-value thresholds. These values should be distinguished from the correlation-based feature counts presented in Fig. 4. However, their subsequent fate differed markedly: while 66% of RP-associated TP candidates were eliminated during biological treatment, only 47% of those detected by MM chromatography were removed. This discrepancy may reflect differences in the sorption behavior of more polar versus less polar compound groups. Conversely, these findings also imply that 34% (RP) and 53% (MM) of AOP-derived TP candidates persisted or even accumulated during biological treatment. Such compounds are particularly relevant from a wastewater management perspective, as they do not originate from industrial influents but are formed during treatment itself. Their detection thus provides valuable insights for plant operators that would be difficult to predict without an NTS. With corresponding substance identifications, this information may ultimately alter the assessment of the effluent’s ecotoxicological potential.

Overall, the data demonstrate that AOP and WWTP processes, which rely on different removal mechanisms (oxidative degradation versus microbiological and sorption-driven elimination), complement each other well. Nevertheless, 24% (RP) and 31% (MM) of initially detected features qualitatively persisted through the combined treatment. This includes features resistant to both AOP and WWTP, as well as AOP-derived TP candidates insufficiently removed during biological treatment. While RP and MM chromatography describe similar trends in this regard, the fraction of persistent MM-related features is significantly larger. A plausible explanation is that MM chromatography detects a broader range of more polar substances, which generally exhibit weaker sorption to activated sludge and therefore lack an effective removal pathway.

Ultimately, these results underline that a substantial fraction of features in this treatment setup cannot be eliminated and would require additional, orthogonal removal mechanisms. Naturally, this group of features warrants particular attention in ecotoxicological investigations.

## Conclusion

This study demonstrates that mixed-mode stationary phases provide a robust alternative to reversed-phase chromatography for non-target screening in complex environmental samples. MM phases deliver comparable data quality to RP, enabling reliable chemometric analyses, while extending coverage into more polar compound domains. Notably, MM chromatography captured a substantially larger number of persistent and AOP-derived transformation products, revealing chemical space that RP alone may miss and highlighting compounds of potential ecotoxicological relevance.

From a practical standpoint, these insights enable the rapid assessment of treatment efficiency and the targeted identification of contaminants that are unlikely to be removed by biological processes alone. Such knowledge supports data-driven optimization of wastewater treatment strategies, for example, by adjusting AOP parameters or implementing additional orthogonal oxidation steps.

In conclusion, MM chromatography does not replace RP but provides a complementary and synergistic extension, broadening chemical characterization and offering alternative perspectives on the fate and transformation of compounds during treatment. Method selection in non-target screening, therefore, critically shapes the chemical information available, with MM opening a window to polar and otherwise underrepresented features.

## Supplementary Information

Below is the link to the electronic supplementary material.

Supplementary file1 (DOCX 2.91 MB)

## Data Availability

The data that support the findings of this study are available from the corresponding author upon reasonable request.
