# Supplementary material for: Comparative non-target screening of advanced oxidation process (AOP)-integrated industrial wastewater treatment using RP and mixed-mode chromatography
Source: Anal Bioanal Chem. 2026 Jul 27;418(21):6943–55. doi: 10.1007/s00216-026-06691-z (PMC13658396; doi:10.1007/s00216-026-06691-z)
Supplement: Supplementary file 1 [file 216_2026_6691_MOESM1_ESM.docx]

Electronic Supplementary Material

**Comparative Non-Target Screening of Advanced Oxidation Process (AOP)-Integrated Industrial Wastewater Treatment using RP and Mixed-Mode Chromatography**

Felix Drees^a,b^, Max Reuschenbach^b,c^, Gerrit Renner***^a,b^ and Torsten C. Schmidt^a,b,d^

*^a^**Instrumental Analytical Chemistry, University of Duisburg-Essen, Universitätsstraße 5, 45141 Essen, Germany*

*^b^Centre for Water and Environmental Research, University of Duisburg-Essen, Universitätsstraße 5, 45141 Essen, Germany*

*^c^LANUK Landesamt für Natur, Umwelt und Klima Nordrhein-Westfalen (LANUK), Leibnizstr. 10, 45659 Recklinghausen, Germany*

*^d^IWW Water Centre, Moritzstrasse 26, 45476 Mülheim an der Ruhr, Germany*

**Corresponding author (gerrit.renner@uni-due.de)*

**Contents**

[Supporting information on HRMS instrumentation	2](#_Toc198893176)

[Data conversion with msConvert	2](#_Toc198893177)

[Quality Control Samples	3](#_Toc198893178)

[MZmine4 parameters	4](#_Toc198893179)

[log K_OW_ of all considered compounds listed in the NORMAN substance database	6](#_Toc198893180)

[Fold-change analysis of concatenated feature lists	6](#_Toc198893181)

[Probability plots PLS-DA analysis	7](#_Toc198893182)

# Supporting information on HRMS instrumentation

SI table 1: API-ESI source settings in positive and negative ionization mode.

| **Atmospheric Pressure Electrospray Ionization Parameters** | | |
| --- | --- | --- |
| **Polarity** | positive | negative |
| **Transfer capillary temperature [°C]** | 320 | 360 |
| **Spray voltage [kV]** | 3.5 | 3.0 |
| **Sheath gas flow rate [a. u.]** | 37 | 40 |
| **Aux gas flow rate [a. u.]** | 15 | 15 |
| **Aux gas heater temperature [°C]** | 50 | 360 |
| **Sweep gas flow rate [a. u.]** | 1 | 0 |
| **S-lens RF level** | 50 | 50 |

SI table 2: Information on full scan MS^1^ parameters.

| **Full‑MS‑Scan Parameters** | | |
| --- | --- | --- |
| **Modus** | positive | negative |
| **Resolution** | 70,000 | 70,000 |
| **AGC target** | 1,000,000 | 1,000,000 |
| **Maximum IT [ms]** | 100 | 50 |
| **Scan range** | 80 to 1000 m/z | 80 to 1000 m/z |

# Data conversion with msConvert

SI table 3: msConvert software settings applied to convert raw chromatographic data from profile mode into .mzML file format.

| **msConvert Options** | |
| --- | --- |
| Binary encoding precision | 64-bit |
| Write index | √ |
| Use zlib compression | √ |
| TPP compatibility | √ |
| **Centroidization** | |
| Filter | Peak Picking |
| Algorithm | Vendor |
| MS Levels | 1 - 2 |
| Min SNR | 0.1 |
| Min peak spacing | 0.1 |

# Quality Control Information

SI table 4: List of target chemicals including information on sum formular and exact mass. Each compound was added in a concentration of 1 µg/L.

| **Analyte** | **Sum formula** | **Exact Mass** | **[M+H]^+^** | **[M−H]^−^** |
| --- | --- | --- | --- | --- |
| L-Valine | C_5_H_11_NO_2_ | 117.07843 | 118.08626 | 116.07170 |
| L-Aspartic acid | C_4_H_7_NO_4_ | 133.03696 | 134.04478 | 132.03023 |
| L-Tyrosine | C_9_H_11_NO_3_ | 181.07334 | 182.08117 | 180.06662 |
| Glycine | C_2_H_5_NO_2_ | 75.03148 | 76.03930 | 74.02475 |
| L-Glutamine | C_5_H_10_N_2_O_3_ | 146.06859 | 147.07642 | 145.06187 |
| L-Proline | C_5_H_9_NO_2_ | 115.06278 | 116.07060 | 114.05605 |
| L-Tryptophane | C_11_H_12_N_2_O_2_ | 204.08933 | 205.09715 | 203.08260 |
| L-Histidine | C_6_H_9_N_3_O_2_ | 155.06893 | 156.07675 | 154.06220 |
| L-Serine | C_3_H_7_NO_3_ | 105.04204 | 106.04987 | 104.03532 |
| L-Lysine | C_6_H_14_N_2_O_2_ | 146.10498 | 147.11280 | 145.09825 |
| L-Alanine | C_3_H_7_NO_2_ | 89.04713 | 90.05495 | 88.04040 |
| L-Cysteine | C_3_H_7_NO_2_S | 121.01920 | 122.02703 | 120.01247 |
| L-Isoleucine | C_6_H_13_NO_2_ | 131.09408 | 132.10191 | 130.08735 |
| L-Leucine | C_6_H_13_NO_2_ | 131.09408 | 132.10191 | 130.08625 |
| L-Phenylalanine | C_9_H_11_NO_2_ | 165.07843 | 166.08626 | 164.07170 |
| L-Methionine | C_5_H_11_NO_2_S | 149.05050 | 150.05833 | 148.04377 |

SI table 5: Retention time reproducibility via mean RT and respective standard deviation.

| **Analyte** | **RT ± SD [s] (Reversed Phase)** | **RT ± SD [s] (Mixed-Mode)** |
| --- | --- | --- |
| Glycine | 62 ± 1.1 | 438 ± 3.2 |
| L-Alanine | 71 ± 1.3 | 472 ± 1.8 |
| L-Proline | 78 ± 1.4 | 512 ± 1.0 |
| L-Serine | 93 ± 1.6 | 496 ± 1.5 |
| L-Aspartic acid | 118 ± 1.2 | 574 ± 2.1 |
| L-Valine | 142 ± 1.5 | 648 ± 2.9 |
| L-Cysteine | 214 ± 1.4 | 690 ± 4.8 |
| L-Glutamine | 286 ± 1.9 | 731 ± 9.1 |
| L-Methionine | 334 ± 1.3 | 756 ± 3.1 |
| L-Histidine | 368 ± 1.4 | 806 ± 1.7 |
| L-Leucine | 421 ± 0.8 | 884 ± 3.3 |
| L-Isoleucine | 447 ± 1.9 | 931 ± 1.6 |
| L-Tyrosine | 536 ± 0.8 | 1048 ± 2.0 |
| L-Phenylalanine | 682 ± 1.1 | 1175 ± 3.2 |
| L-Tryptophane | 894 ± 0.3 | 1386 ± 2.0 |
| L-Lysine | 965 ± 1.7 | 1515 ± 4.2 |

The retention times of the QC standards remained highly stable throughout the analytical sequence for both chromatographic methods. Overall, RP chromatography showed slightly lower retention time variability compared to the mixed-mode approach. With the exception of L-glutamine in mixed-mode chromatography, all retention time deviations remained within a low single-digit second range and well within the retention time tolerances applied during MZmine feature alignment.

SI table 6: Matrix effects [%] and corresponding standard deviations (SD) of the 16 amino acid quality control standards determined for the reversed-phase (RP) chromatographic method across different wastewater-related matrices. Values represent relative signal responses compared to solvent-based standards. Median values summarize the overall matrix-related signal behavior across all analytes for each matrix type.

| **Reversed Phase** | | | | | | | | |
| --- | --- | --- | --- | --- | --- | --- | --- | --- |
| **Analyte** | **Influent** | **SD** | **Effluent** | **SD** | **BFO** | **SD** | **AAO** | **SD** |
| Glycine | 63.7 | 19.4 | 81.6 | 14.8 | 58.9 | 21.1 | 74.2 | 13.7 |
| L-Alanine | 69.1 | 16.8 | 86.3 | 13.5 | 64.8 | 18.9 | 79.4 | 12.6 |
| L-Proline | 78.6 | 14.9 | 94.7 | 11.3 | 73.8 | 16.4 | 88.9 | 12.8 |
| L-Serine | 74.3 | 15.7 | 89.8 | 12.6 | 69.5 | 17.2 | 84.6 | 13.1 |
| L-Aspartic acid | 76.1 | 14.8 | 91.2 | 12.9 | 71.1 | 16.7 | 86.3 | 13.4 |
| L-Valine | 84.8 | 13.1 | 101.4 | 10.7 | 79.7 | 14.6 | 96.8 | 11.8 |
| L-Cysteine | 91.7 | 11.6 | 108.9 | 9.8 | 86.4 | 13.2 | 103.8 | 10.9 |
| L-Glutamine | 96.4 | 12.3 | 112.7 | 10.1 | 91.2 | 13.5 | 107.6 | 11.1 |
| L-Methionine | 82.6 | 14.2 | 99.8 | 11.1 | 77.9 | 15.8 | 93.4 | 12.2 |
| L-Histidine | 57.4 | 21.6 | 73.8 | 16.9 | 61.2 | 23.4 | 69.1 | 15.8 |
| L-Leucine | 52.8 | 18.7 | 68.9 | 15.4 | 55.1 | 20.3 | 64.7 | 14.2 |
| L-Isoleucine | 61.7 | 17.3 | 77.5 | 13.6 | 58.4 | 19.1 | 72.8 | 14.9 |
| L-Tyrosine | 70.9 | 16.1 | 88.7 | 12.7 | 67.2 | 17.4 | 83.5 | 13.2 |
| L-Phenylalanine | 66.3 | 17.8 | 84.9 | 13.9 | 63.7 | 18.6 | 80.2 | 14.1 |
| L-Tryptophane | 73.4 | 15.4 | 91.6 | 12.3 | 69.8 | 16.7 | 87.3 | 12.9 |
| L-Lysine | 49.6 | 22.8 | 66.7 | 17.6 | 53.1 | 24.3 | 62.8 | 18.4 |
| **Median** | **72.6** | **15.9** | **89.3** | **12.7** | **68.4** | **17.3** | **83.9** | **13.1** |

SI table 7: Matrix effects [%] and corresponding standard deviations (SD) of the 16 amino acid quality control standards determined for the mixed-mode (MM) chromatographic method across different wastewater-related matrices. Values represent relative signal responses compared to solvent-based standards. Median values summarize the overall matrix-related signal behavior across all analytes for each matrix type.

| **Mixed-Mode** | | | | | | | | |
| --- | --- | --- | --- | --- | --- | --- | --- | --- |
| **Analyte** | **Influent** | **SD** | **Effluent** | **SD** | **BFO** | **SD** | **AAO** | **SD** |
| Glycine | 76.8 | 16.2 | 91.9 | 11.6 | 71.4 | 18.3 | 84.7 | 12.8 |
| L-Alanine | 81.2 | 14.1 | 96.8 | 11.4 | 76.1 | 15.7 | 89.8 | 12.1 |
| L-Proline | 89.5 | 12.7 | 104.1 | 9.6 | 84.1 | 13.5 | 99.2 | 10.7 |
| L-Serine | 84.1 | 13.2 | 99.4 | 10.8 | 79.6 | 14.1 | 94.1 | 11.6 |
| L-Aspartic acid | 85.7 | 12.8 | 101.2 | 10.9 | 81.3 | 13.8 | 95.6 | 11.3 |
| L-Valine | 94.6 | 11.2 | 111.6 | 8.7 | 89.2 | 12.1 | 106.3 | 9.8 |
| L-Cysteine | 101.3 | 9.7 | 118.4 | 7.9 | 96.1 | 10.8 | 113.7 | 8.9 |
| L-Glutamine | 106.8 | 9.8 | 123.1 | 7.6 | 101.7 | 10.9 | 117.9 | 8.4 |
| L-Methionine | 93.7 | 12.1 | 109.8 | 9.2 | 88.4 | 12.9 | 104.6 | 10.1 |
| L-Histidine | 63.5 | 19.8 | 79.6 | 14.7 | 66.8 | 21.1 | 76.4 | 15.2 |
| L-Leucine | 59.6 | 17.9 | 74.2 | 13.8 | 61.3 | 18.6 | 71.8 | 14.1 |
| L-Isoleucine | 67.9 | 16.4 | 82.1 | 12.8 | 64.2 | 17.5 | 78.6 | 13.5 |
| L-Tyrosine | 74.8 | 14.9 | 91.4 | 11.8 | 71.6 | 15.8 | 87.9 | 12.4 |
| L-Phenylalanine | 72.1 | 16.3 | 89.3 | 12.6 | 69.4 | 17.1 | 85.8 | 13.2 |
| L-Tryptophane | 81.2 | 14.2 | 98.9 | 10.8 | 77.4 | 15.1 | 94.1 | 11.6 |
| L-Lysine | 58.9 | 20.3 | 75.1 | 15.2 | 62.4 | 21.7 | 71.3 | 16.1 |
| **Median** | **81.2** | **14.5** | **97.9** | **11.1** | **76.8** | **15.4** | **91.9** | **11.9** |

Matrix effects were observed for all investigated wastewater-related matrices and chromatographic methods, with median signal responses generally ranging between approximately 70 and 120% relative to solvent-based standards. Overall, mixed-mode chromatography showed slightly higher signal responses and lower variability compared to the reversed-phase setup. Influent and BFO samples exhibited stronger matrix-related signal suppression and variability than effluent and AAO samples, reflecting the increased matrix complexity of untreated wastewater samples.

SI figure 2: Combined normalized signal intensities of the internal standards monitored throughout the study. Signal intensities were normalized to the mean response of each analyte to enable comparison of relative signal stability. Certain standards are highlighted due to high variability.

Overall, the normalized signal intensities remained centered around unity, with most standards fluctuating within approximately **0.85-1.15** over the analytical sequence. For the majority of analytes, signal variability was low to moderate, with relative standard deviations generally in the range of **5-10%**. More pronounced deviations were limited to a small number of standards, most notably **L-isoleucine**, which showed transient signal increases up to approximately **1.7** relative to its mean response. Apart from these isolated deviations, the normalized responses indicated stable batch performance and adequate long-term signal stability.

SI table 8: Relative standard deviations (RSDs) of the internal standards calculated from their signal intensities across the analytical sequence. RSD values were used to assess internal standard signal stability and batch reproducibility.

| **Analyte** | **RSD [%]** | **Analyte** | **RSD [%]** |
| --- | --- | --- | --- |
| Glycine | 6.8 | L-Methionine | 5.9 |
| L-Alanine | 5.1 | L-Histidine | 10.1 |
| L-Proline | 6.4 | L-Leucine | 9.2 |
| L-Serine | 7.8 | L-Isoleucine | 24.6 |
| L-Aspartic acid | 6.1 | L-Tyrosine | 5.9 |
| L-Valine | 5.8 | L-Phenylalanine | 7 |
| L-Cysteine | 4.8 | L-Tryptophane | 4.8 |
| L-Glutamine | 7.4 | L-Lysine | 8.4 |

Signal reproducibility was generally high across the analytical sequence, with most amino acid standards showing RSD values below 10%. The majority of standards ranged between 4.8 and 9.2% RSD, indicating stable signal response and good batch reproducibility. L-histidine showed a slightly elevated RSD of 10.1%, while L-isoleucine exhibited the highest variability with 24.6% RSD, consistent with the transient signal spikes observed in the normalized signal intensity plot (SI figure 2).

# MZmine4 parameters

SI table 9: MZmine4 settings for feature extraction.

| **Workflow** | **Parameter** | **Mass detection** | **Chromatogram building** | | **Smoothing (Loess)** | **Chromatogram deconvolution** | **^13^C Isotope filter** | **Join aligner** | **Gap filling (peak finder)** | **Feature list blank subtraction** |
| --- | --- | --- | --- | --- | --- | --- | --- | --- | --- | --- |
| Peak Detection | Mass detector | Factor of lowest signal |  | |  |  |  |  |  |  |
|  | Noise factor | 5.0 |  |  |  |  |  |  |  |  |
|  | MS level | 1.0 |  |  |  |  |  |  |  |  |
|  | Min. group size in # of scans |  | 10 | |  |  |  |  |  |  |
|  | Group intensity threshold |  | 0 | |  |  |  |  |  |  |
|  | Min height intensity |  | 0 | |  |  |  |  |  |  |
|  | m/z tolerance |  | 0.0025 Da or 5 ppm | |  |  |  |  |  |  |
|  |  | | | |  |  |  |  |  |  |
|  | Algorithm |  | | | | Local minimum feature resolver |  |  |  |  |
|  | Chromatographic threshold [%] |  |  |  |  | 90.0 |  |  |  |  |
|  | Minimum search range RT/Mobility [abs] |  |  |  |  | 0.05 |  |  |  |  |
|  | Minimum relative height [%] |  |  |  |  | 0.0 |  |  |  |  |
|  | Minimum absolute height |  |  |  |  | 10,000 |  |  |  |  |
|  | Min ratio of peak top/edge |  |  |  |  | 2.0 |  |  |  |  |
|  | Peak duration range [min] |  |  |  |  | 0.0-10.0 |  |  |  |  |
|  | Min # of data points |  |  |  |  | 10 |  |  |  |  |
|  | m/z Tolerance (intra-sample) |  | | | | | 0.001 Da or 5 ppm |  |  |  |
|  | Retention time tolerance [s] |  |  |  |  |  | 5.0 |  |  |  |
|  | Maximum charge |  |  |  |  |  | 1 |  |  |  |
|  | Representative isotope |  |  |  |  |  | Single most intense |  |  |  |
| Alignment, Completion and blank correction | m/z Tolerance |  | |  | | | | 0.0025 Da or 5 ppm |  |  |
|  | Weight for m/z tolerance |  |  |  |  |  |  | 0.5 |  |  |
|  | Retention time tolerance [absolute, min] |  |  |  |  |  |  | 0.4 |  |  |
|  | Weight for RT |  |  |  |  |  |  | 0.5 |  |  |
|  | Require same charge state |  |  |  |  |  |  | yes |  |  |
|  | Intensity tolerance [%] |  |  |  |  |  |  |  | 15 |  |
|  | m/z Tolerance |  |  |  |  |  |  |  | 0.0025 Da or 5 ppm |  |
|  | Retention time tolerance [min] |  |  |  |  |  |  |  | 0.15 |  |
|  | # Minimum scans |  |  |  |  |  |  |  | 1 |  |
|  | Minimum # of detection in blanks |  | | | | | | | | 2 |
|  | Fold change increase [%] |  |  |  |  |  |  |  |  | 200 |

The parameters for feature extraction were selected based on the developers’ recommendations and prior experience with LC-HRMS-based NTS workflows, with the aim of maintaining a comparatively permissive feature extraction strategy. In particular, low group intensity thresholds were intentionally applied to minimize the risk of excluding low-abundance yet potentially relevant features during chromatogram building. Consequently, the initial feature lists also contained a substantial proportion of low-intensity and potentially blank-associated signals.

To compensate for this intentionally low-selective extraction strategy, subsequent quality control steps were applied, including blank subtraction and data quality scoring (DQS), which evaluated signal consistency and feature reliability across the analytical sequence. The distribution of sample-to-blank intensity ratios across all detected features is shown in SI figure 3. Most features exhibited intensity ratios substantially above the applied 2-fold (200%) threshold, whereas approximately 20.4% of all detected features fell below this threshold and were therefore considered blank-related and removed. The broad distribution above the threshold further indicates that the selected cutoff did not represent an excessively stringent filtering criterion but rather excluded predominantly low-ratio background-associated features.

The same processing workflow was applied consistently across both stationary phases and ionization modes, resulting in four separate feature lists. Depending on the evaluation scenario, these lists were either processed individually or combined across chromatographic modes.

SI figure 3: Distribution of sample-to-blank intensity ratios (N = 150,708 MS features) and effect of applying a 2-fold (200%) threshold for feature filtering. The histogram shows the number of features within each intensity ratio interval, calculated as the median feature intensity in samples divided by the median feature intensity in blanks. Features with ratios below 2-fold were considered blank-related and removed, resulting in the exclusion of 30,800 features (20.4%). Features with ratios ≥2-fold were retained for further data processing, corresponding to 119,908 features (79.6%).

**Feature Counts throughout Data Processing depending on Chromatography and Ionization Mode**

For each condition, the number of features was initially high and reduced after blank subtraction:

- **C_18__pos**: 50,427 features initially, reduced to 35,206 after blank subtraction.
- **C_18__neg**: 14,191 features initially, reduced to 11,287 after blank subtraction.
- **MM_pos**: 61,206 features initially, reduced to 52,036 after blank subtraction.
- **MM_neg**: 24,884 features initially, reduced to 21,379 after blank subtraction.

Furthermore, injection peak-associated features were removed during the first 3 minutes, leading to the following results:

- **C_18__pos**: 10,278 features removed, resulting in 24,928 features remaining.
- **C_18__neg**: 4,842 features removed, leaving 6,445 features.
- **MM_pos**: 11,113 features removed, leaving 40,923 features.
- **MM_neg**: 6,181 features removed, leaving 15,198 features.

# log K_OW_ of all considered compounds listed in the NORMAN substance database

SI figure 4: Violin and box plot illustrating the distribution of log K_ow_ values for considered compounds listed in the NORMAN substance database. The violin plot displays the shape of the data distribution, where wider sections reflect a higher frequency of values within that range. The overlaid box plot shows key statistical metrics, including the median and interquartile range (IQR), with box edges corresponding to the first and third quartiles.

# Fold-change analysis of concatenated feature lists

SI figure 5: Volcano plots representing differential feature distribution originating from the AOP and WWTP sampling series. Volcano plots illustrate the distribution of detected features based on statistical significance and fold-change. The x-axis represents the log₂ fold-change, indicating the magnitude of differences between two conditions, while the y-axis shows the negative log₁₀(p-value), reflecting statistical significance. Features with significantly increased abundance in one condition appear on the right (red), while those with decreased abundance are on the left (green). The dashed horizontal line denotes the significance threshold. Non-significant features are displayed in gray, with those showing no variation in their fold-change being regarded as persistent during the respective process (orange).

# Probability plots PLS-DA analysis

SI figure 6: Probability plots of the PLS-DA classification results for four predefined sample classes. Each subplot pair shows the class prediction probabilities for the respective data source: left = reversed-phase (RP), right = mixed-mode (MM). Elevated probabilities for the correct class indicate clear separation between groups.

SI table 10: Cross-validation performance of the PLS-DA models developed from RP- and mixed-mode chromatographic datasets. The table summarizes sensitivity, specificity, classification error, root mean square error of cross-validation (RMSECV), and coefficient of determination (R²CV) for the investigated sample classes.

| **Sample class** | **Chromatography** | **Sensitivity (CV)** | **Specificity (CV)** | **Classification Error (CV)** | **RMSECV** | **R²CV** |
| --- | --- | --- | --- | --- | --- | --- |
| B-AOP | RP | 0.992 | 0.986 | 1.1 % | 0.239 | 0.83 |
| B-AOP | MM | 1.000 | 1.000 | 0.0 % | 0.248 | 0.83 |
| A-AOP | RP | 1.000 | 0.968 | 1.6 % | 0.280 | 0.81 |
| A-AOP | MM | 1.000 | 0.993 | 0.36 % | 0.252 | 0.89 |
| Influent | RP | 0.972 | 1.000 | 1.4 % | 0.409 | 0.63 |
| Influent | MM | 0.860 | 0.954 | 9.3 % | 0.413 | 0.62 |
| Effluent | RP | 1.000 | 1.000 | 0.0 % | 0.355 | 0.87 |
| Effluent | MM | 0.940 | 0.974 | 4.3 % | 0.381 | 0.81 |
